# Novel Intronic Mutations Introduce Pseudoexons in DMD That Cause Muscular Dystrophy in Patients

**DOI:** 10.3389/fgene.2021.657040

**Published:** 2021-04-16

**Authors:** Xinguo Lu, Chunxi Han, Jiahui Mai, Xianping Jiang, Jianxiang Liao, Yanqi Hou, Di Cui

**Affiliations:** ^1^Department of Neurology, Shenzhen Children’s Hospital, Shenzhen, China; ^2^Department of Pathology, Shenzhen Children’s Hospital, Shenzhen, China; ^3^Running Gene Inc., Beijing, China

**Keywords:** cDNA analysis, target DNA sequencing, *DMD*, Duchenne muscular dystrophy, Becker muscular dystrophy, movement disorder

## Abstract

**Background:** Duchenne muscular dystrophy (DMD) and Becker muscular dystrophy (BMD) are two subtypes of muscular dystrophy diseases caused by pathogenic mutations in the *DMD* gene. Until now, more than 4,600 disease-causing mutations in *DMD* have been reported. However, only 33 mutations were deep intronic, cases with this type of mutations were limited.

**Methods:** In this study, we used a combination of complementary DNA (cDNA) and target DNA sequencing analysis in addition to conventional whole-exome sequencing (WES).

**Results:** Three novel hemizygous mutations IVS11 + 17811C > G (c.1331 + 17811C > G), IVS21 + 3252A > G (c.2803 + 3252A > G) and IVS40 + 362A > G (c.5739 + 362A > G) were identified in DMD patients, while a reported hemizygous mutation IVS62-285A > G (c.9225-285A > G) was found in the BMD patient. These *DMD* mutations lead to pseudoexon insertions, causing the generation of truncated and dysfunctional dystrophin.

**Conclusion:** This study defines three novel and one reported intronic mutations, which can result in DMD/BMD. We also emphasize the need to combine WES and cDNA-based methods to detect the variant in the very large *DMD* gene in which the mutational spectrum is complex.

## Introduction

Duchenne muscular dystrophy (DMD) and Becker muscular dystrophy (BMD) are X-linked recessive hereditary diseases caused by mutations in the *DMD* gene (OMIM^∗^300377). Nonsense or frame-shift mutations of the *DMD* gene lead to the generation of prematurely truncated proteins, which result in DMD (Cau et al., 2012). The motor development of DMD patients is generally normal at birth or early infant. However, most patients presented progressive muscular atrophy in childhood and lost their ability to walk between ages of 10 and 12 years (Magri et al., 2011). They commonly died of heart or respiratory failure at around 30 years old (Singh et al., 2018). Compared to DMD, BMD is less severe. It is usually caused by in-frame mutations, with the expression of partially functional dystrophin (Gloss et al., 2016). Other causes include alternative initiation sites, exon deletions, and differences in intronic deletion breakpoints as well as genetic and environmental modifiers (Ciafaloni et al., 2016). BMD occurs juvenile or adult, patients present with a wide range of clinical manifestations from quite mild symptoms to a slightly less severe DMD-like condition (Beggs et al., 1991; Bushby et al., 1993).

The *DMD* gene (2.4 million bases, including 79 exons and eight promoters) is one of the largest genes in the human genome. Multiple types of mutations in the *DMD* gene have been identified, including large deletion/insertion, single base mutation, and intron mutation. Large deletion/insertion and point mutation in the coding region of *DMD* can be detected by Sanger sequencing and whole-exome sequencing (WES). However, the detection of deep intronic mutations is challenging due to the huge genomic size of *DMD* gene with introns account for 99%. Deep intronic mutations may generate a new splicing site, retaining a part of the intron to the coding region of the transcript, and resulting in the splicing of a pseudoexon into mRNA (Ikezawa et al., 1999; Beroud et al., 2004; Gurvich et al., 2008; Keegan, 2020). Recently, RNA sequencing has been identified as a powerful clinical diagnostic tool for the diagnosis of neuromuscular disease, which focuses on detecting deep intronic mutations and splice changes (Gonorazky et al., 2019). However, RNA sequencing has not been widely used in diagnosis due to the high cost. In comparison, cDNA sequencing can be considered as a back-up choice for transcript analysis.

In this study, a combination of complementary DNA (cDNA) and target DNA sequencing analysis in addition to conventional WES was used to identify deep intronic mutations in *DMD* gene. Four cases with pseudoexon insertions (caused by identify deep intronic mutations) in the *DMD* gene were described. Three patients displayed typical DMD phenotypes, and one patient had the clinical symptoms of BMD. All four mutations located over 200 bp from exons, which lead to pseudoexon insertions, causing the generation of truncated and dysfunctional dystrophin. With novel mutations identified in *DMD*, we activated pseudoexon inclusion in patients who were suspected to have DMD or BMD.

## Materials and Methods

### Patient Enrollment

Cases reported in this paper were screened from 1,440 clinical diagnosed BMD/DMD patients of Shenzhen Children’s Hospital. Patients with normal *DMD* exon sequence and immunohistochemistry analysis of muscle biopsy that demonstrated altered or absent dystrophin expression were selected to perform the cDNA analysis. Four mutations were finally identified in four unrelated patients. Informed consent was obtained from all patients.

### Histopathological and Immunochemical Analysis

The 50 mg skeletal muscle of patients was obtained and processed for histopathological and immunochemical analysis. For histopathological analysis, muscle specimens were fixed in 10% formalin, embedded in paraffin, prepared into 10 μm sections, then routinely stained with Hematoxylin and Eosin (HE). For immunohistochemistry (IHC) staining, muscle specimens were cryopreserved in liquid nitrogen immediately after biopsies. Tissue blocks were cut into 8 μm cryosections and then stained against N-terminal (DYS3), rod-domain (DYS1) and C-terminal (DYS2) epitopes (Novocastra). Experimental results of the patients 1–3 were similar. Thus, only biopsy findings of patients 2 (DMD patient) and 4 (BMD patient) have been chosen to be analyzed.

### cDNA Analysis

Total RNA was isolated from frozen muscle tissues of all four patients using the RNA extraction kit (TIANGEN) according to the manufacturer’s recommended protocol. cDNA was synthesized using the Superscript III first-strand synthesis kit (Invitrogen) with random hexamer primers. A series of cDNA primers were designed spanning alone the exon 1–79 of the *DMD* gene using Oligo 7 (Supplementary Table 1). All primers were synthesized based on the wild type sequence at both ends of each exon. The mRNA sequences were obtained by amplifying the cDNA fragments of four patients. Target fragments were PCR amplified using 10 pmol of each primer, 0.2 mmol dNTPs, 1 × PCR buffer (TAKARA), 1.5 U Taq DNA polymerase (TAKARA), and 2 μL of cDNA products in a total volume of 25 μL. Incubation was 94°C, 5 min for denaturation followed by 12 cycles at 94°C, 30 s; 60°C, 30 s; 72°C, 45 s; and then 35 cycles at 94°C, 30 s; 55°C, 30 s; 72°C, 45 s with a last elongation step for 72°C 10 min. Sequence insertions were determined by Sanger sequencing and compared with the reference DMD sequence (GenBank accession number NM_004006.2). For each detected pseudoexon, the origin was determined by the BLAST program to identify the corresponding intronic fragment.

### DNA Analysis

In order to define insertion breakpoints, peripheral blood samples of four patients and their family members were obtained. DNA was extracted using the extraction kit (TIANGEN) according to the manufacturer’s recommended protocol. The primers were designed spanning 200 bp flanking sequences of the insertion sequence in the genomic DNA using Oligo 7 (Supplementary Table 1). Target fragments were PCR amplified, purified and Sanger sequenced. The insertion breakpoints were determined by aligning the sequencing results with the reference sequence.

### Splice-Site Strength Scoring Methods

We used the following methods to analyze the strength of the pseudoexon splice sites: (1) the deep residual neural network SpliceAI was used to predict both acceptor and donor gains, scores of which were evaluated as 0.2 (high recall), 0.5 (recommended), and 0.8 (high precision) (Jaganathan et al., 2019); (2) the first-order Markov model was used to examine and weigh the only dependencies between adjacent positions within the splice-site sequence; (3) the Maximum Entropy Model was used to take into account dependencies between adjacent and non-adjacent positions; and (4) the Shapiro and Senapathy position-weight matrix was used to reflect the degree of conservation at each position of the consensus 5′ and 3′ splice motifs^1^ (Gurvich et al., 2008). For methods (2)-(4), a higher score indicates a greater probability of the corresponding sequence being recognized as a splice site but does not represent the absolute predictive value.

## Results

### Clinical Phenotypes

**FIGURE 1:**
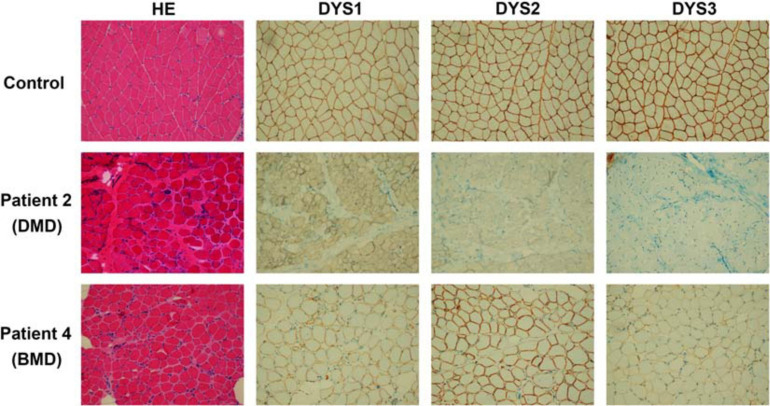
Results of histopathological and immunochemical analysis. The DMD patient (patient 2), BMD patient (patient 4), and a healthy control are shown. Patient 2 presents the absence of dystrophin expression. While patient 4 shows dystrophin reduction.

All four unrelated patients showed clinical features consistent with a dystrophinopathy (Table 1). Patient 1 was an 8-year-old boy, who displayed elevated aminotransferase and muscle weakness of both lower limbs at the age of 3 years. Further examination revealed calf hypertrophy and Gower’s sign. The serum CK concentration was extremely high (18,086 IU/L), and a muscle biopsy immunohistochemical staining showed absent dystrophin expression in the fibers. Patient 2 was a 5-year old boy with delayed motor development. He could not crawl at 10 months, walked with imbalance gait at the 13 months, and began to walk independently at 15 months, but was prone to falls. At the age of 3 years, he displayed multiple lower limb abnormalities including gastrocnemius pseudohypertrophy, weak tendon reflexes in both knees, and abnormal fatigue after exercise. His serum CK was high (18,090 IU/L). And the muscle biopsy results were similar with that of patient 1 (Figure 1). Patient 3 was a 9-year-old boy, who presented with high serum CK (153,000 IU/L) and hypokinesia at the age of 4 years. Histological staining of muscle biopsy indicated severe fibrosis, fat substitution, and absent dystrophin expression. All these three patients had typical clinical manifestations including lower limb proximal muscle weakness, Gower’s sign and gastrocnemius pseudohypertrophy. Their serum aspartate aminotransferase (AST), alanine aminotransferase (ALT), and creatine kinase (CK) were significantly increased before their 5 years old. Also, there was little dystrophin expression in the muscle fiber membranes. Thus, these three patients were diagnosed with DMD based on these typical clinical features.

**TABLE 1:** Detailed characteristics of identified DMD/BMD patients.

	**Patient 1**	**Patient 2**	**Patient 3**	**Patient 4**
Age (y)	8	5	9	13
Gender	M	M	M	M
Age at onset (y)	3	3	4	9
Waddling gait	++	+	++	+
Calf muscle pseudohypertrophy	++	++	++	+
Hyporeflexia	Mild	Mild	Mild	–
Hypotonia	Mild	Mild	Mild	–
Gowers sign	+	+	+	–
Serum creatine kinase (IU/L, normal 25-192 IU/L)	18,086	18,090	15,300	6,470
Serum alanine transaminase (IU/L, normal 40 IU/L)	361	324	296	154
Serum aspartate aminotransferase (IU/L, normal 40 IU/L)	315	310	260	178

Patient 4 was a 13-year-old boy who had no obvious symptoms of muscle weakness before the age of 8 years. When he was 9 years old, he started to feel weak after exercise. The serum AST, ALT, and CK were relatively high (178, 154, and 6,470 IU/L, respectively). He displayed lower limb muscle weakness but still could walk independently. Muscle biopsy revealed reduced dystrophin expression in muscle fibers (Figure 1). Hematoxylin and Eosin (H&E) staining also revealed areas of fibrosis and degenerating fibers. He was finally diagnosed as BMD (Table 1).

### mRNA Transcript Isoforms

For each patient, previous WES analysis using peripheral blood samples indicated normal genotypes of the coding regions and the splicing sites of their *DMD* genes. In order to perform further molecular diagnosis, muscle tissues were obtained from all four patients for mRNA extraction and RT-PCR sequencing. Inserted fragments were detected in the cDNA of each patient (referring to NC_000023.11) with at least one premature stop codon, which was predicted to truncate the protein translation. A 120 bp inserted fragment was detected in the cDNA of patient 1 (g.32626322-g32626441), which contained three identified premature stop codons (g.32626329, g.32626380, and g32626416) and would result in the expression of mutant protein with 444 normal sequenced amino acids. An 88 bp inserted fragment was detected in patient 2’s cDNA (g.32481668-g32481755). It had one premature stop codon (g.32481689), which would truncate the protein shortly after 935 correct amino acids. Patient 3’s cDNA had a 78 bp insertion (g.32342773-g32342850) with three premature stop codons (g.32342779, g.32342782, and g.32342818), which might encode truncating protein with 1,914 normal sequenced amino acids. While Patient 4 had a 58 bp inserted fragment (g.31261306-g31261363) with a premature stop codon (g.31261349). The mutated gene might express the protein with 3,076 correct amino acids and a short messy tail (Table 2).

**TABLE 2:** Pseudoexon insertion mutations in four patients.

**Patient no.**	**Diagnosis**	**Pseudoexon derivation/size**	**Mutation**		
			**DNA**	**RNA**	**Protein**
1	DMD	Intron 11; 120 nt	c.1331 + 17811C > G	c.1331_1332ins1331 + 17691_1331 + 17811	p.Asn444Lysfs
2	DMD	Intron 21; 88 nt	c.2803 + 3252A > G	c.2803_2804ins2803 + 3164_2803 + 3251	p.Ile935Asnfs
3	DMD	Intron 40; 78 nt	c.5739 + 362A > G	c.5739_5740ins5739 + 284_5739 + 361	p.Glu1914Metfs
4	BMD	Intron 62; 58 nt	c.9225-285A > G	c.9224_9225ins9224 + 62235_9224 + 62292	p.His3076Valfs

*DMD, Duchenne muscular dystrophy; BMD, Becker muscular dystrophy.*

### Genetic Information

**FIGURE 2:**
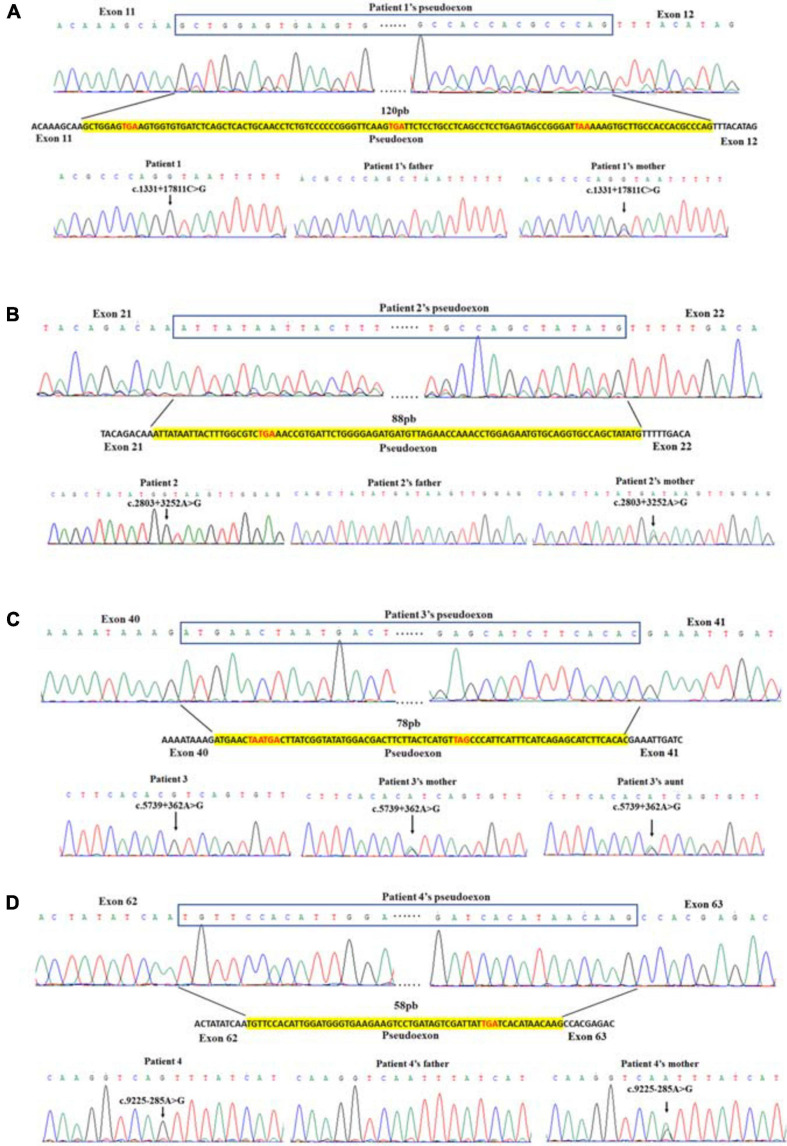
Identification of pseudoexon and disease-causing mutation in each patient. Junctions of exon and pseudoexon were shown. Pseudoexons were squared in cDNA sequence, separated from exon junctions. Insertion sequence was highlighted in yellow. Pseudoexon sizes (bp) were indicated above the insertion sequence, and premature stop codons were represented in red letters. The disease-causing deep intronic variant of each patient was displayed under the nucleotide sequence, together with sequencing results of their family pedigree. Arrows indicated the site of variant.

**FIGURE 3:**
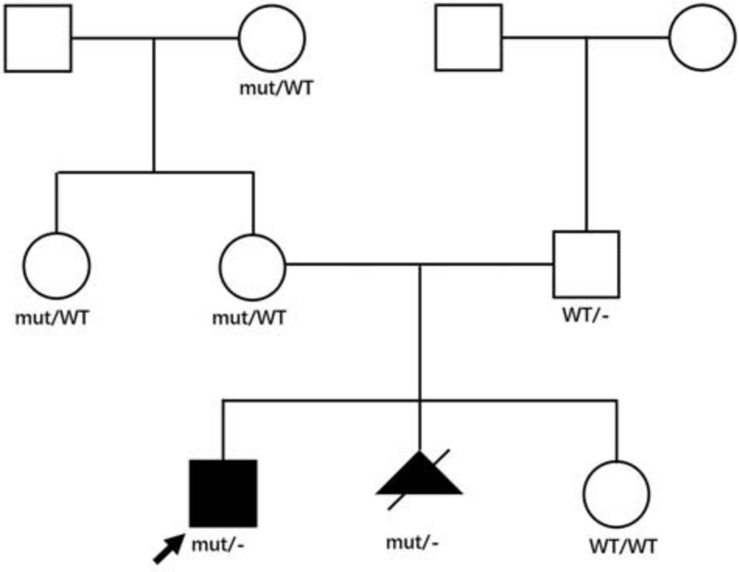
Family pedigree of patient 3. Genotypes in the patient 3’s family were displayed in the pedigree. Affected patients were displayed as black symbols and healthy members were marked in white symbols. *mut* represented the mutant X allele with c.5739 + 362A > G, WT represented the wild type X allele, – represented Y allele. The proband was indicated with an arrow. The second pregnancy of the proband’s mother was aborted after the same genotype was identified, and the genotype of her third pregnancy was normal.

We analyzed the sequences around the inserted fragments to investigate the underlying cause of the pseudoexon insertion (referring to NM_004006.2). Three novel hemizygous single nucleotide variants c.1331 + 17811C > G, c.2803 + 3252A > G, and c.5739 + 362A > G were identified at the splice donor sites in DMD patients, resulting in a 120 bp insertion in intron 11, an 88 bp insertion in intron 21 and a 78 bp insertion in intron 40, respectively. A reported disease-causing mutation c.9225-285A > G was found at the splice acceptor site in a BMD patient, leading to a 58 bp insertion in intron 62 (Tuffery-Giraud et al., 2003; Table 2 and Figure 2). Besides, the co-segregation analysis was also performed on the family members of these patients, results of which indicated that all four patients’ intron mutations were inherited from their mothers. Additionally, heterozygous mutation of c.5739 + 362A > G was also detected in other family members of patient 3 (Figure 3).

### Pathogenicity Predictions

SpliceAI was used to predict both acceptor and donor gains. As shown in Table 3, SpliceAI predicted that all variants detected in patients create new splice donor sites, leading to pseudoexon inclusion. Other prediction tools Shapiro and Senapathy (S&S) consensus splice-site weight matrix, first-order Markov models (MM), and Maximum Entropy Model (ME) were also employed to value the relative strength scores of the splice sites created by the deep intronic mutations. The strength of both the splice donor site and splice acceptor site was very strong according to results calculated by these three independent algorithms except the splice donor site in patient 3 (Table 3), indicating high probabilities of abnormal splicing in patients 1, 2, and 4. Although the splicing possibility of the mutant splice donor site of patient 3 was lower than others, the strong splice acceptor site enhanced the splicing efficiency, resulting in the generation of pseudoexon insertion.

**TABLE 3:** Mutation sites, potential donor splice site sequences and splice scores.

**Patient**	**5′ Splice-site (donor) sequence and scores**					**3′ Splice-site (acceptor) sequence and scores**				
	**Sequence and mutation site**	**SpliceAI**	**SS**	**ME**	**MM**	**Sequence**	**SpliceAI**	**SS**	**ME**	**MM**
1	cagGtaatt (C > G)	0.47	9.17	8.55	7.72	agttttgttctttcacccaggct	0.00	11.07	6.89	7.89
2	atgGtaagt (A > G)	0.75	10.67	11.1	10.1	atatctgaatattattgcagatt	0.00	3.72	6.03	6.97
3	cacGtcagt (A > G)	0.90	5.7	5.37	3.59	tttgtgtatctttgttccagatg	0.00	10.34	10.7	11.52
4	aaggtcaGt (A > G)	0.76	8.19	8.68	8.34	tgtcggtgtcctttctgtagtgt	0.00	8.74	8.31	9.05

*Capital letter indicated mutation site. SpliceAI predicts both acceptor and donor gains. SpliceAI scores were evaluated as 0.2 (high recall), 0.5 (recommended), and 0.8 (high precision).*

## Discussion

With the development and application of molecular diagnostic techniques, various types of mutations have been found in the *DMD* gene. Most of the variants could be easily detected by multiplex ligation-dependent probed amplification (MLPA) or WES. The deep intronic variants, however, were left undetected. Since the first deep intronic disease-causing mutation in *DMD* reported by Ikezawa et al. (1999), only 33 deep intronic mutations have been detected by array CGH (Khelifi et al., 2011) or cDNA analysis (Zaum et al., 2017). Cases with this type of mutations were limited.

In this study, we reported different single nucleotide substitutions in *DMD* gene of four patients, which induced pseudoexon inclusions with in-frame stop codons in mature mRNA of *DMD*, causing the truncation of dysfunctional dystrophin. Patients 1–3 presented with typical clinical manifestations including lower limb proximal muscle weakness, Gower’s sign, gastrocnemius pseudohypertrophy, high levels of serum AST, ALT, CK, and little dystrophin expression in the muscle fiber membranes. They were finally diagnosed with DMD. Patient 4, however, displayed lower limb muscle weakness but still could walk independently. The serum AST, ALT, and CK were relatively high and the muscle biopsy revealed reduced dystrophin expression in muscle fibers. He was finally diagnosed as BMD. What should be mentioned is that patient 4 should also be diagnosed with DMD due to the stop codon in the pseudoexon. However, compared with patients 1–3 reported in our study, patient 4 had milder clinical manifestations. He didn’t have hyporeflexia or hypotonia, and the CK level (6,470 IU/L) was lower than other three patients (18,086, 18,090, and 15,300 IU/L). The results of muscle biopsy were relatively normal than other patients (Figure 1). The age at onset was older (9 years) and he still could walk independently at the age of 13 years. Therefore, patient 4 was finally diagnosed with BMD.

The clinical manifestations of these four patients with DMD/BMD were attributed to the aberrantly spliced mRNA of *DMD*, while the WES results were negative. cDNA analysis could suggest the location of variants deep in introns, expanding the range of genomic detection and improving the accuracy of molecular diagnosis. What is more, gels and western blots should also have been performed to detect the fractionation of the RT-PCR amplicons, to see if non-sense mediated decay contributed to reduced levels of dystrophin mRNA, to analyze the amount normal splicing transcripts and the exact expression of dystrophin. However, these experiments could not be performed for the lack of muscle tissues (all had been used up). So we could not do more experiments. We speculated that multiple transcripts were expressed in patients 1–2 due to the cDNA sequence traces shown in Figures 2A,B, among which the mRNA containing a pseudoexon accounted for the majority. Normal protein should have been expressed in patient 4, but at a quite low level that could not be detected by Sanger sequencing (Figure 2D). This could explain his milder phenotype, as previously reported by Tuffery-Giraud (Tuffery-Giraud et al., 2003).

Previous studies showed that intronic mutations located outside of splice site sequences that affect splicing may account for 25% of pathogenic mutations, and this ratio can be even higher for some particular genes or exons (Padgett, 2012). So far, only 33 deep intronic disease-causing mutations in *DMD* have been reported in HGMD (Stenson et al., 2017), which is even less than 0.05% of the total reported pathogenic mutations. In ClinVar, the percentage of pathogenic deep intronic variants was less than 0.6% among all recorded *DMD* variants (Landrum et al., 2014). In this paper, three reported novel deep intronic variants verified the contribution of targeted RNA-Seq for clinical diagnosis and extended the genomic spectrum of *DMD*. In addition, the disease-causing mutations in 2–7% patients with typical DMD/BMD symptoms were still not identified (Dent et al., 2005; Okubo et al., 2016). These patients could benefit from the molecular diagnosis through cDNA analysis combined with DNA sequencing to verify the possible pathogenic mutations located deeply in introns.

Technically speaking, deep intronic variants could also be identified by full-length sequencing of *DMD* gene. However, in addition to the higher cost, hundreds of deep intronic variants, most of which are begin or the variant of undetermined significance, would be difficult to be annotated from full-length sequencing or the data interpretation. Positioning the ranges of variants by cDNA analysis and identifying the sites of mutations by target DNA sequencing would greatly reduce the cost. Pathogenic deep intronic variants were not rare, which had already been identified in more than 75 disease-causing genes (Vaz-Drago et al., 2017). Considering the less charge and a high labor-intensive of these technologies, the combined sequencing strategy deserves extensive spread to promote the diagnosis of DMD and many other diseases. It should be mentioned that in our study, simple fragment size analysis of the PCR products were not conducted (agarose gel analysis), making our study imperfect. Such an initial screen by fragment size would complement our approach to detecting pseudoexon insertions. What should also be mentioned is that perhaps RNA could not be undertaken in most conditions that had been used for sectioning and storage. The amount of muscle tissue used in our study is substantial, while only small amount of mRNA could be obtained in our study. We have to use a total of 47 cycles to amplify cDNA of the *DMD* gene, which consisted of 12 cycles with the annealing temperature 60°C, and a 35-cycle with the annealing temperature 55°C. If the amount of mRNA is sufficient, there is no need to amplify the cDNA with too many cycles, which might generate non-specific amplifications and interfere with sequencing.

## Conclusion

In conclusion, we identified three novel and one reported deep intronic disease-causing mutations in *DMD* genes, which can cause DMD/BMD in patients. We also emphasized the need to combine WES and cDNA-based methods to detect the variant in the very large *DMD* gene in which the mutational spectrum is complex. With novel mutations found in *DMD*, we activated pseudoexon inclusion in patients suspected to have DMD or BMD.

## Data Availability Statement

The raw data supporting the conclusions of this article will be made available by the authors, without undue reservation.

## Ethics Statement

The studies involving human participants were reviewed and approved by Ethics Committee of Shenzhen Children’s Hospital. Written informed consent to participate in this study was provided by the participants’ legal guardian/next of kin.

## Author Contributions

XL designed the project, set the goal, and wrote the discussion of the manuscript. CH supervised the project and reviewed the manuscript. JM enrolled patients, arranged the clinical evaluation, and drafted the clinical portion of the manuscript. XJ performed clinical tests and record data. JL performed the biopsy staining and pathological evaluation. YH and DC drafted the genetic portion of the manuscript, reviewed and revised the manuscript. All authors read and approved the final manuscript.

## Conflict of Interest

YH and DC were employed by the company Running Gene Inc. The remaining authors declare that the research was conducted in the absence of any commercial or financial relationships that could be construed as a potential conflict of interest.

## Footnotes

1 http://genes.mit.edu/burgelab/maxent/Xmaxentscan_scoreseq.html

## Abbreviations

DMD: Duchenne muscular dystrophy
BMD: Becker muscular dystrophy
WES: whole-exome sequencing
AST: aspartate aminotransferase
ALT: alanine aminotransferase
CK: creatine kinase
H&E: hematoxylin and eosin
PCR: polymerase chain reaction
DHPLC: denaturing high performance liquid chromatography
RT-PCR: reverse transcription-polymerase chain reaction
S&S: Shapiro and Senapathy
MM: Markov Models
ME: maximum entropy model
CGH: comparative genome hybridization
HGMD: Human Gene Mutation Database.
