# Supplementary material for: Novel Intronic Mutations Introduce Pseudoexons in DMD That Cause Muscular Dystrophy in Patients
Source: Front Genet. 2021 Apr 16;12:657040. doi: 10.3389/fgene.2021.657040 (PMC8085517; doi:10.3389/fgene.2021.657040)
Supplement: Supplementary file 1 [file Table_1.docx]

**Table S1. Primer sequences used in this study**

| **Target region** | **Forward primer** | **Reverse primer** |
| --- | --- | --- |
| 1. cDNA analysis | | |
| exon 1-7 | ACTGGAGCAATAAAGTTTGAAGAAC | CTGGCCTATGACTATGGATGAGA |
| exon 6-10 | AAGATTCTCCTGAGCTGGGTC | CTCCATCAATGAACTGCCAAA |
| exon 9-13 | GGCTGCTTATGTCACCACCTCT | TTCATCAACTACCACCACCATG |
| exon 12-17 | AGTACAACAACATAAGGTGCTTCA | AATCCACAGTAATCTGCCTCTTC |
| exon 16-21 | CACCACTCAGCCATCACTAACA | TAGCCGGTTGACTTCATCCTTA |
| exon 20-24 | CAGAACAACATCATCGCTTTCTAT | AAAACATCAACTTCAGCCATCC |
| exon 23-27 | AAATTGAGGGACGCTGGAA | GTGGAGCTTGAGCTATGACACTA |
| exon 27-30 | CTGTAAGCCTCCAGAAAGAT | AGCTGCGTCCACCTTGTCTG |
| exon 30-34 | TAATCCAGGAGTCCCTCACAT | CAGGCAACTTCAGAATCCAAA |
| exon 34-37 | ATTGTCCCGTAAGATGCGAAAG | AGCTCTGAGATTTGGGGCTCTA |
| exon 37-41 | ATACGCCCAAAGGTGGACTC | CCTCAGCTTGCCTACGCACT |
| exon 40-44 | AAAATTAGCCAGCCTACCTG | GTCAAATCGCCCTTGTCG |
| exon 43-47 | TATTCATAGCAAGAAGACAGCAGCAT | GCACGGGTCCTCCAGTTTCA |
| exon 46-50 | GATAACATTGCTAGTATCCCACTT | CTAGGTCAGGCTGCTTTGC |
| exon 49-53 | AAAACCAGCCACTCAGCC | AGGTGTTCTTGTACTTCATCCC |
| exon 52-55 | AAAACAAGACCAGCAATCAAG | GAGTCTTCTAGGAGCCTTTCC |
| exon 55-59 | AAGTTTCTTGCCTGGCTTACA | TCCTCAGCCTGCTTTCGTAG |
| exon 58-63 | GCAGCCTTTGGAAGGACTA | CAGGCGGTCATAAATAGTGG |
| exon 60-67 | GGACTTTGGTCCAGCATCTCA | GCAACTTCACCCAACTGTCTTG |
| exon 65-70 | CGGGACGAACAGGGAGGAT | TCTGCACTGGCAGGTAGCC |
| exon 69-75 | ATTATGACATCTGCCAAAGC | GTTCGTGCTGCTGCTTTA |
| exon 74-79 | GATGATGAACATTTGTTAATCCAG | AAAACCATGCGGGAATCA |
| 1. Genomic DNA analysis | | |
| pseduoexon 11 | CAACTCGCAGGTTTGATACA | TACTTTTAAATGCACGCCAT |
| pseduoexon 21 | TAGTTATGACAAAGCCAGTGTT | TTCGGAAATGTCTACTGCTC |
| pseduoexon 40 | TTCTTGTTTCTATTAACGTTCTC | TTTAGAGGGTAAGTGAATAAGTT |
| pseduoexon 62 | TAGCATTTACAATGAGTAGTCAAGAA | GAAATAATGGGAAGAAGGAAGT |
